# The clinical biochemistry laboratory computer system as a simple calculator: a program in MUMPS

**DOI:** 10.1155/S1463924685000086

**Published:** 1985

**Authors:** T. G. Pellar, N. Rawal, A. R. Henderson

**Affiliations:** Department of Clinical Biochemistry University Hospital (University of Western Ontario) PO Box 5339, Postal Stn. A Ontario London N6A 5A5 Canada


Journal of Automatic Chemistry, Volume 7, Number 1 (January–March 1985), pages 38–42

### The clinical biochemistry laboratory computer system as a simple calculator: a program in MUMPS

**T. G. Pellar, N. Rawal and A. R. Henderson***
*Department of Clinical Biochemistry, University Hospital (University of Western Ontario), PO Box 5339, Postal Stn. A, London, Ontario, Canada, N6A 5A5*

### Introduction

The hand-held calculator is ubiquitous in analytical laboratories. Its major use is for simple arithmetical manipulations, such as totalling the test numbers processed during a shift or converting a series of absorbance values to concentrations from the absorbance value of a standard. The same facility is not so readily available on a laboratory computer system. Therefore we have the frequent paradox of a technologist sitting in front of a video terminal of the main computer system reaching for a hand-held calculator!

If access to the programming environment of the main laboratory computer can be gained, then, to calculate the value of the expression, say, 1·7 × 2·9 requires, in BASIC, the statement PRINT 1·7*2·9 or, in MUMPS, WRITE 1·7*2·9†. By contrast, with a hand-held calculator the same manipulation requires only two keystrokes using either conventional algebraic operations or reverse Polish notation. Therefore the hand-held calculator remains both more convenient and efficient than the main laboratory computer for simple but very common calculations.

We now describe a program written in MUMPS which can be called on the main laboratory computer system and which is as convenient and efficient as a hand-held calculator when used for simple calculations.

### Materials and methods

The laboratory computer system is a 32-bit Data General Eclipse MV/6000 with one megabyte of core memory (Data General [Canada] Ltd, Mississauga, Ontario). We used the XL-87H video display terminal (Cybernex Ltd, Ottawa, Ontario) which emulates a Hazeltine terminal. The computer operating and programming system was written in a dialect of MUMPS (Medical Information Technology Inc., Cambridge, Massachusetts) [3].

* *Corresponding author.*

† *In certain versions of BASIC, PRINT can be replaced by P. [1] and in ANS MUMPS, WRITE can be replaced by W [2]. Therefore the expression then requires three or four keystrokes (in addition to the data) to process.*

The operating system allows direct access to the MUMPS programming mode by means of passwords. Operating in this direct mode does not interfere with the main laboratory system or its data-base.

*Table 1. The HELP routine. This narrative outlines the use of each option. Each section (DECIMALS, ARITHMETIC and so on) is displayed sequentially on the screen by pressing ENTER.*

**DECIMALS**

Several of the available routines request the user to define how many decimal places are to be displayed in the answer. Precision for the four operations of addition, subtraction, multiplication and division is maintained so that an accuracy of 16 significant figures is possible. Consider the following examples for which 15 decimals were requested.

1/3 = ·333333333333333
100/·3 = 333·333333333333300
2/3 = ·666666666666667
200/·3 = 666·666666666666700

The first and third examples display an answer to 15 decimals, rounded off appropriately. The second and fourth examples both contain 16 significant figures but are padded on the right with zeroes to fit the requested format of 15 decimal places. Be aware of this feature when dealing with a large number of decimal places.

**ARITHMETIC**

The arithmetic option allows you to enter an arithmetic expression which the computer will evaluate for you. The arithmetic operators that the system recognizes are as follows:

+ addition
− subtraction
* multiplication
/ division

Expressions may be input as in the following general examples:

100 + 126·55
0·76–100·2
20·0*·73
1265/1·667

NOTES: Spaces are not allowable within the expression. One operator is allowable per expression. Preceding zeroes may be dropped e.g. ·76 is the same as 0·76.

**STRING ADDITION**

The string addition option allows the user to input a list of numbers to be totalled without the need to input the + operator between each number. With this option simply input each

*Table 1.*

number, press <ENTER>, and then a final <ENTER> will produce the total.

```
Example: BEGIN ENTRY  => 100
                         10·1
                         ·57
                         1000·3
               TOTAL  =  1110·97
```

**COMPOUND ARITHMETIC**

This option allows you to do multiplication operations using the result of previous calculations such as a hand-held or desk-top calculator. Begin by entering your first operation. The result will appear below waiting for your next operation. A sample operation follows, with entries underlined:

```
BEGIN ENTRY  => 313*·6 = 187·80
             => 187·80/2 = 93·90
             => 93·90 + 100 = 193·90
             => 193·90
BEGIN ENTRY  =>
```

The initial operation is a number followed by an operator followed by a number (the same syntax rules apply as for ARITHMETIC). Subsequent operations on this result are input as an opertor followed by a number. In the example, the expression (((313·0*0·6)/2) + 100) has been evaluated.

**MULTIPLY BY CONSTANT**

This option allows you to enter a constant which will be used to multiply subsequent numbers by. The routine works as follows:

```

      ENTER CONSTANT:    2·5

         BEGIN ENTRY:    => 100

the computer responds with –

         BEGIN ENTRY:    => 100*2·5 = 250·0

                         =>

```

**DIVIDE BY CONSTANT**

This option allows you to enter a constant which will be used to divide into subsequently entered numbers. This routine works as follows:

```

      ENTER CONSTANT:    −6·3

         BEGIN ENTRY:    => 200

the computer responds with –

         BEGIN ENTRY:    => 200/−6·3 = −31·75

```

*Table 2. The Introductory Sequence. This routine presents the initial menu of options—see text. On program line CAL + 1, ^COUNTER is a global variable used to count the number of accesses to this program; it is updated (the count is indexed by 1) on calling the program.*

```
CAL #  ; INTRODUCTORY PROG FOR CALCULATOR 180984 TGP
       W # ! S BF="BAD FORMAT", $U (^COUNTER)
       W !?5 F I=1:1:69 W $C(96)
       F I=1:1:3 W ?5,$C(96),?75,$C(96),!
       W ?5,$C(96),?30,"C A L C U L A T O R",?75,$C(96),!
       F  I=1:1:3 W ?5,$C(96),?75,$C(96),!
       W ?5 F I=1:1:71 W $C(96)
       W !
       F  I=1:1:3 W ?5,$C(96),?75,$C(96),!
       W ?5,$C(96),?30,"OPTIONS:",?75,$C(96),!
       W ?5,$C(96),?75,$C(96),!
       W ?5,$C(96),?30,"ARITHMETIC",?75,$C(96),!
       W ?5,$C(96),?30,"STRING ADDITION",?75,$C(96),!
       W ?5,$C(96),?30,"COMPOUND ARITHMETIC",?75,$C(96),!
       W ?5,$C(96),?30,"MULTIPLY BY CONSTANT",?75,$C(96),!
       W ?5,$C(96),?30,"DIVIDE BY CONSTANT",?75,$C(96),!
       W ?5,$C(96),?30,"HELP",?75,$C(96),!
       F  I=1:1:2 W ?5,$C(96),?75,$C(96),!
       W ?5 F I=1:1:71 W $C(96)
LA     R !!?25,"CALCULATOR OPTION: ",ANS W:ANS="?" ! S CNT I 'ANS K Q
LC     S  CNT= $U(CNT),L=$T(OPT+CNT) I 'L W:ANS'="?" *7,"NOT FOUND":3 G LA
       I  ANS="?" W !?25,$P(L;2) G LC
       I  $E($P(L;2),1,$L(ANS))+ANS W $E(P(L;2),$L(ANS)+1,$L($P(L;22))),! X $P(L;3) G LA
       G LC
OPT    ;LIST OF CALCULATOR OPTIONS
       ;ARITHMETIC;C CAL
       ;STRING ADDITION;C CAL2
       ;COMPOUND ARITHMETIC;C CAL@CSA
       ;MULTIPLY BY CONSTANT;C CAL2@CLF
       ;DIVIDE BY CONSTANT;C CAL2@DLF
       ;HELP;C CA1
       G L
```

*Table 3. The Calculator Functions, part 1. This program contains the routines called by the menu selection for Arithmetic and Compound Arithmetic (table 2).*

```

CAL    ;CALCULATOR FUNCTIONS: ARITHMETIC 081184 TGP

       K (BF) C CU@SF

CLB    K (SD,SR,BF) R !!?25,"EXPRESSION: ",EXP:300 Q:'EXP

       C CU@EXTRACT I X=400 W BF:3 G CLB

       I LS'?1NN I LS'?"-"1NN I LS'?1NN"."1NN I LS'?"-"1NN"."1NN I LS'?"."1NN F:3 G CLB

       I RS'?1NN I RS'?"-"1NN I RS'?1NN"."1NN I RS'?"-"1NN"."1NN I RS'?"."1NN F:3 G CLB

       I (($M($E(EXP,E,$L(EXP))*1)=0)&($F(EXP,"/")'="")) W "NO DIVISION BY ZERO 3 G CLB

       S "RE="_"$M("_EXP_")" I $E(RE,1,1)="-" S_"RE="_"$M("_RE_"-"_SR_","_SD)" W " = ",RE G CLB

       E S _"RE="_"$M("_RE_"+"_SR_","_SD_")" W " = ",RE G CLB

CSA    ;COMPOUND ARITHMETIC 081184 TGP

       K (BF) C CU@SF

SA     K (SD,SR,BF) W !!!?25,"BEGIN ENTRY "

SB     R ?38,"= ",EXP:300 Q:'EXP

       C CU@EXTRACT I X=400 W BF:3 G SA

       I LS'?1NN I LS'?"-"1NN I LS'?1NN"."1NN I LS'?"-"1NN"."1NN I LS'?"."1NN W F:3 G SA

       I RS'?1NN I RS'?"-"1NN I RS'?1NN"."1NN I RS'?"-"1NN"."1NN I RS'?"."1NN W F:3 G SA

       I (($M($E(EXP,E,$L(EXP))*1)=0)&($F(EXP,"/")'="") W "NO DIVISION BY ZERO 3 G SA

       S _"RE="_$M("_EXP_")" I $E(RE,1,1)="-" S _"RE-"-"$M("_"-"_SR_","_SD )" W " = ",RE,!!

       E S _"RE="_"$M("_RE_"+"_SR_","_SD_")" W " = ",RE,!!

SC     W ?38,"=> ",RE R FE:300 G:'FE SA

       S FC=$E(FE,1,1),RC=$E(FE,2,$L(FE))

       I FC'="+" I FC'="-" I FC'="*" I FC'="/" W BF:3,! G SC

       I RC'?1NN I RC'?"-"1NN I RC'?1NN"."1NN I RC'?"-"1NN"."1NN I RC'?"."1NN W F:3 G SC

       I (($M(RC*1)=0)&(FC="/")) W "NO DIVISION BY ZERO":3,! G SC

       S _"RE"="_"$M("_RE_FE_")" I $E(RE,1,1)="-" S_"RE="_"$M("_RE_"-"_SR","_S")" W " = ",RE,!! G SC

       E S _"RE="_"$M("_RE_"+"_SR_","_SD_")" W " = ",RE,!! G SC

```

*Table 4. The Calculator Functions, part 2. This program contains the routines called by the menu selection for String Addition, Multiply and and Divide by Constant (table 2).*

```

CAL2   ;CALCULATOR FUNCTIONS: STRING ADDITION 091184 TGP

       K (BF) W !!!?25, "BEGIN ENTRY"

A      R ?38,"=> ",NUM G: 'NUM B

       I NUM'?1NN I NUM'?"-"1NN I NUM'?1NN"."1NN I NUM'?"-"1NN"."1NN I NUM'?" N W BF:3,! G A

       S TOTAL=$M(TOTAL+NUM) W ! G A

B      W !?34, "TOTAL = ",TOTAL

       R !!?16,"<PRESS ENTER TO CONTINUE OR ANY OTHER KEY TO EXIT>",ANS Q:ANS G CAL2

CLF    ;MULTIPLY BY CONSTANT

       K (BF) C CU@SF

FA     K (SD,SR,BF) R !!?25,"ENTER CONSTANT: ",FAC:300 Q:'FAC

       I FAC'?1NN I FAC'?"-"1NN I FAC'?"-"1NN"."1NN I FAC'?1NN"."1NN I FAC'?"." N W BF:3 G FA

       W !!?25, "BEGIN ENTRY "

FB     R ?38,"=> ",NU:300 G:'NU FA

       I NU'?1NN I NU'?"-"1NN I NU'?"-"1NN"."1NN I NU'?1NN"." 1NN I NU'?"."1NN W F:3,! G FB

       W "*", FAC," = " S_"RE="_"$M("_FAC_"*"_NU_")" I $E(RE,1,1)="-" S _"RE="_ M("_RE_"-"_SR_","_SD_")" W RE,!!

       G FB

E      S -"RE="_"$M("_RE_"+"_SR_","_SD_")" W RE,!!G FB

G      FB

DLF    ;DIVIDE BY CONSTANT

       K (BF) C CU@SF

DA     K (SD,SR,BF) R !!?25,"ENTER CONSTANT: ",FAC:300 Q:'FAC

       I FAC'?1NN I FAC'?"-"1NN I FAC'?"-"1NN"."1NN I FAC'?1NN"." 1NN I FAC'?". "N W BF:3 G DA

       I $M(FAC*1)=0 W "CANNOT DIVIDE BY ZERO!":3 G DA

       W !!?25,"BEGIN ENTRY "

DB     R ?38,"=> ",NU:300 G:'NU DA

       I NU'?1NN I NU'?"-"1NN I NU'?"-" 1NN."1NN I NU'?1NN"."1NN I NU'?"."1NN W F:3,! G DB

       W "/",FAC," = " S _"RE="_"$M("_NU_"/"_FAC_")" I $E(RE,1,1)="-" S _"RE="-"M("_RE_"-"_SR_","_SD_")" W RE,!! G

       DB

       E S_"RE="_"$M("_RE_"+"_SR_","_SD_")" W RE,!! G DB

```

*Table 5. The Numeric Format and Decimal Place programs.*

```

CU       ;CALCULATOR UTILITY FUNCTIONS 091184 TGP

         ;REQUEST DECIMALS

SF       R  !?25,"HOW MANY DECIMALS? 2 // ",SD:300

         I  'SD S SD=2

         I  (15 SD)! (0 SD)!(SD'?1NN) W "WHOLE NUMBERS 0 to 15 ONLY":3 G SF

         F  I=1:1:SD S SR=SR_0

         S  SR="."_SR_5 Q

EXTRACT ; FIND RIGHT AND LEFT SIDE OF EXPRESSION

         I  $F(EXP, "-")=2 S B=$F(EXP, "-",2)

         E  S B=$F(EXP,"-")

         I  B="" S B=100

         S  A=$F(EXP,"+") I A="" S A=100

         S  C=$F(EXP,"*") I C="" S C=100

         S  D=$F(EXP,"/") I D="" S D=100

         S  X=A+B+C+D I X=100 Q

         S  E=(((A&B)&C)&D)

         S  LS=$E(EXP,1,(E-2)),RS=$E(EXP,E,$L(EXP)) Q

```

### The program

On calling the program the options are presented. These are:

ARITHMETIC
STRING ADDITION
COMPOUND ARITHMETIC
MULTIPLY BY CONSTANT
DIVIDE BY CONSTANT
HELP.

The options are selected by entering the first letter of the required option. The HELP routine is shown in table 1. It requires 4·2 Kbyte of memory. This narrative gives examples of the type of allowable calculations which have found to be commonly performed in this laboratory setting; advice is also provided regarding the number of decimal places that can be obtained.

The ARITHMETIC option allows the use of the common operators (addition, subtraction, multiplication and division) with two terms. If a multi-term manipulation is required it can be done, after paying due attention to the order of operations, by using the COMPOUND ARITHMETIC option. A common manipulation in an analytical laboratory is the conversion of a series of absorbance values to analyte concentrations using a factor derived from the absorbance of a standard. This is readily achieved using the MULTIPLY BY CONSTANT option. The converse operation, DIVIDE BY CONSTANT, is also available. The final option, STRING ADDITION, allows the addition (tally) of a sequence of numbers without the inclusion of the '+' operator. This routine is therefore more efficient than a hand-held calculator. The tally operation appears to be the most common one used in the University Hospital's laboratory.

These programs are contained in two modules—the Introductory Sequence (table 2) and the Calculation Function (tables 3, 4 and 5). Together these programs required 4·7 Kbyte of memory. For readers unfamiliar with the MUMPS language, a list of the common commands and functions used may be of value:

*Commands*

| | |
|---|---|
| CALL (C] | — sends program execution to the specified program |
| DO (D) | — sends program execution within a program |
| FOR (F) | — initiates repeated execution of following commands |
| GOTO (G) | — moves program execution to a specified line |
| IF (I) | — permits conditional execution of a line |
| KILL (K) | — removes all variables except those in brackets |
| QUIT (Q) | — terminates the action of a DO, CALL or FOR command |
| READ (R) | — outputs specified carriage control instructions and/or string and accepts data to be placed in a specified variable. It is also used as a timing device to sign the program out if no response has been given within 300 s (see line CLB, table 3). |
| SET (S) | — assigns a value to a variable |
| WRITE (W) | — outputs variables, strings or carriage control commands |
| EXECUTE (X) | — executes code which has been stored in a string. |

*Functions*

| | |
|---|---|
| $CHAR ($C) | — returns the ASCII character specified |
| $EXTRACT ($E) | — returns from a specified string the characters at a specified location |
| $FIND | — returns the position of a substring within a string |

| | |
|---|---|
| $LENGTH ($L) | — returns the number of characters in a specified string |
| $MULTI-PRECISION ($M)* | — facilitates floating point arithmetic |
| $PIECE ($P) | — returns from a specified string a piece as determined by specified markers |
| $TEXT ($T) | — returns the code from a specified program line |
| $UPDATE ($U)* | — returns an updated numeric valued variable. |

*Operators*

| | |
|---|---|
| _ | — indirection or concatenation (underscore symbol) |
| ? | — pattern matching. |

The ease with which this program handles numeric manipulations is due to the use of 'indirection' [4]. MUMPS regards all data as variable length character strings. An arithmetic expression may be input as a string and become the value of a variable such as SET EXP = '176 + 22'. If this is followed by WRITE _EXP, 198 would be printed. The indirection operator causes the variable immediately following (in this case EXP, which equals '176 + 22') to be evaluated. Another more complicated example is:

WRITE _A_'+'_B_'+'_C (where A = 100, B = 20, C = 30) which is printed as 150. Within the code, the trailing underscores serve as concatenators and the indirection operators cause the concatenated results to be evaluated before it is written.

* *$M and $U are not ANS MUMPS functions; they are unique MIIS, vendor-specific, functions.*

### Discussion

The regular advertizing of programs for microcomputers that can emulate a hand-held calculator is clear evidence that there is a general need for a calculator capability on microcomputers. This present program provides only the four common arithmetical operators, as these are, by far, the ones most needed. Other operators, such as square root, logarithms, exponents and trigonometric functions, are not currently available but are being considered for inclusion in future rewrites of this program.

The almost universal acceptance of the calculator program in the University Hospital's laboratory shows that a pressing need has been met, particularly in areas using numeric data. Program details for the HELP routine may be obtained by writing to the authors.

### References

1. Lien, D. A., *The BASIC Handbook*, 2nd edn (Compusoft Publishing, San Diego, 1981), 272.
2. Sherertz, D., ANS MUMPS Programmers' Reference Manual 1983 (MUMPS Users' Group, College Park, 1983), 91.
3. MIIS Reference Manual, Version S.MIIS.R-II-3.1 (Medical Information Technology, Inc., Cambridge, 1983), 1.
4. Walters, R. F., Bowie, J. and Wilcox, J. C., MUMPS Primer, Revised (MUMPS Users' Group, College Park, 1982), 83.

**SAC 86/3rd BNASS**

This is to be a combined conference incorporating SAC 86 and the Third Biennial National Atomic Spectroscopy Symposium; it will be held from 20 to 26 July 1986 at the University of Bristol, UK. It will consist of invited lectures, contributed papers, posters and workshops. Submitted papers are needed by 31 October 1985: titles should be sent to:

*Miss P. E. Hutchinson, Analytical Division, Royal Society of Chemistry, Burlington House, London W1V 0BN.*

A series of update courses is planned for the conference; also an exhibition of scientific equipment and books.

